# Hemodynamic changes in the temporalis and masseter muscles during acute stress in healthy humans

**DOI:** 10.1007/s00421-023-05349-3

**Published:** 2023-11-17

**Authors:** Anas Rashid, Silvestro Roatta

**Affiliations:** Lab of Integrative Physiology, Department of Neuroscience “Rita Levi Montalcini”, University of Torino, Corso Raffaello 30, 10125 Torino, Italy

**Keywords:** Blood flow, Oxygenation, Masseter, Temporalis, Muscle, Stress

## Abstract

**Purpose:**

Autonomic control of orofacial areas is an integral part of the stress response, controlling functions such as pupil dilatation, salivation, and skin blood flow. However, the specific control of blood flow in head muscles during stress is unknown. This study aims to investigate the hemodynamic response of temporalis and masseter muscles in response to five different stressors.

**Methods:**

Sixteen healthy individuals were subjected to a randomized series of stressors, including cold pressor test, mental arithmetic test, apnea, isometric handgrip, and post-handgrip muscle ischemia, while in the sitting posture. Finger-pulse photoplethysmography was used to measure arterial blood pressure, heart rate, and cardiac output. Near-infrared spectroscopy was used to measure changes in tissue oxygenation and hemoglobin indices from the temporalis and masseter muscles.

**Results:**

All stressors effectively and significantly increased arterial blood pressure. Tissue oxygenation index significantly increased in both investigated head muscles during mental arithmetic test (temporalis: 4.22 ± 3.52%; masseter: 3.43 ± 3.63%) and isometric handgrip (temporalis: 3.45 ± 3.09%; masseter: 3.26 ± 3.07%), suggesting increased muscle blood flow. Neither the masseter nor the temporalis muscles evidenced a vasoconstrictive response to any of the stressors tested.

**Conclusion:**

In the different conditions, temporalis and masseter muscles exhibited similar hemodynamic patterns of response, which do not include the marked vasoconstriction generally observed in limb muscles. The peculiar sympathetic control of head muscles is possibly related to the involvement of these muscles in aggressive/defensive reactions and/or to their unfavorable position with regard to hydrostatic blood levels.

## Introduction

Autonomic control of extracranial areas of the head is an integral and relevant component of social behavior and includes control of pupil size, salivation, skin blood flow, etc. (Benarroch 2020). Regarding blood flow control, several systems including parasympathetic, sympathetic, and trigeminal sensory systems are possibly involved, with a complex interplay of dilatory and constrictory actions (Izumi 1999; Ishii et al. 2011). While there is an agreement about the possible sympathetic dilatory action in facial skin, e.g., during flushing (Drummond and Lance 1987), whether a peculiar sympathetic control also concerns head muscles is unknown.

Interest in the control of muscle blood flow also comes from clinical studies. Altered masticatory muscle perfusion is implicated in numerous painful disorders afflicting head and orofacial regions (Curtis et al. 2012; Dawson et al. 2015), whereby inadequate delivery of oxygen and nutrients occurring during and after muscle contraction leads to muscle damage and functional impairment (Delcanho et al. 1996). In this respect, the control of blood flow exerted by the sympathetic nervous system is of particular interest due to its general constrictory action oriented to limit blood flow, to both resting and working muscles (Thomas and Segal 2004; Roatta et al. 2011) which has been implicated in pathophysiological mechanisms behind musculoskeletal disorders (Koltzenburg 1997; Jänig and Häbler 2000; Hämmig 2020) and also in the orofacial area (Melis et al. 2002). However, only few investigations have been carried out on the control of blood flow in masticatory muscles in physiological (e.g., acute stress exposure) (Kim et al. 1999; Ning et al. 2016) or pathological (e.g., chronic tension-type headache) (Langemark et al. 1990) conditions.

The control of sympathetic outflow may be highly differentiated to different body regions and numerous reports described an increase rather than a decrease in blood flow or tissue oxygenation in the masseter muscle during experimental stress (Delcanho et al. 1996; Maekawa et al. 1998; Kim et al. 1999). By the simultaneous recording of tissue oxygenation in the masseter and biceps brachii muscles (Rashid and Roatta 2022), we recently confirmed these observations, during exposure to different sympathetic activation tests: significant tissue oxygenation decreased in the biceps and increased in the masseter muscle. The reason for a different hemodynamic response of the masseter muscle to stress is unclear, and it is also unknown whether this preferential dilatory rather than constrictory response is specific to the masseter muscle or is shared by other head muscles. In fact, we hypothesized that this feature could characterize head muscles in general, due to the unfavorable hydrostatic gradient that affects the head, compared to trunk and limb muscles, in humans in the erect posture.

Most studies of masticatory muscle perfusion have been confined to the masseter, a jaw-closing muscle, whereas other head muscles have been little considered for hemodynamic investigations. We recently validated the possibility of using near-infrared spectroscopy (NIRS) on the temporalis muscle. In particular, the possibility was excluded that interference from the deeper brain tissue could affect the measurement (Rashid and Roatta 2023). Thus, this present study aims to test the hypothesis that the dilatory response to acute stress observed in the masseter muscle is shared by the temporal muscle. NIRS monitoring will be simultaneously performed on temporal and masseter muscles but, differently from our previous study (Rashid and Roatta 2022), the subjects will maintain the sitting rather than the supine position, in order to test the secondary hypothesis that the hemodynamic response to stress is independent of current head-heart hydrostatic gradients. In fact, in our previous study, subjects were investigated in the supine position so that both the masseter and biceps muscles were positioned at heart level (Rashid and Roatta 2022), and it is presently unknown whether these responses are affected by body posture.

## Methods

### Subject and ethical approval

Sixteen healthy university students participated in this study (8 males, 8 females; age: 25 ± 4 years; weight: 69 ± 15 kg; height: 173 ± 10 cm). The investigation was carried out according to the Declaration of Helsinki and was approved by Comitato di Bioetica dell'Università degli Studi di Torino (Protocol # 60195). All subjects gave their written informed consent before participation.

### Monitoring equipment and measurements

Finger-pulse photoplethysmography (CNAP^®^ Monitor, CNSystems Medizintechnik GmbH, Graz, Austria) was used to measure arterial blood pressure (ABP), heart rate, and cardiac output. Calibration of ABP was periodically performed using a regular pneumatic cuff at the left arm.

A custom-made device based on film sensors (FlexiForce A201 Tekscan, Boston, MA, USA) was used to measure handgrip force (Pertusio and Roatta 2023) and visual feedback has been provided to each subject to maintain constant force during isometric handgrip.

Electromyographic signals (Quattro, OT Bioelettronica SRL, Torino, Italy; Gain 1200; Bandwidth 10–500 Hz) were recorded from right anterior temporal and right superficial masseter muscles by means of pairs of surface electrodes (FIAB SpA, Florence, Italy, inter-electrode distance 2.3 cm, inter-electrode axis parallel to the orientation of the muscle fibers, ground electrode stuck to the right ear), to detect possible involuntary muscle contraction throughout the experiment.

NIRS (NIRO-200NX, Hamamatsu Photonics, Hamamatsu, Japan) was used to measure changes in tissue oxygenation index (TOI, %) and tissue hemoglobin index (THI, a.u.). The device has two probes: one was placed over the left anterior temporal muscle (Rashid and Roatta 2023) and the other over the left superficial masseter muscle (Rashid and Roatta 2022).

### Experimental procedures

Experiments were conducted in a silent, light-attenuated, and temperature controlled (23–25 °C) room, with a subject sitting in a comfortable chair. All signals reached a stable condition, then a 10-min baseline was recorded, after which volunteers completed a sequence of five different tasks in a randomized order, described below, except post-handgrip muscle ischemia which immediately followed the isometric handgrip. All volunteers were periodically reminded to relax their head muscles, especially masticatory muscles.

i. Cold pressor test: The participant was asked to immerse and maintain for 1 min the right hand into a bucket filled with cold water (10°C), and after the end of the task to rate the peak pain level experienced during the task using a visual analogue scale (from 0, no pain; to 10, worst imaginable pain).
ii. Mental arithmetic test: he participant was asked to progressively subtract the odd numbers (1, 3, 5 and so) from 1000 for 2 min while writing the outcome on the paper. One operator standing behind the subject monitored the outcome and compared it to a table reporting the correct answers, promptly asking the subject to repeat the calculation in case of error.
iii. Apnea: The participant was asked to hold their breath for 40 sec to maintain apnea.
iv. Isometric handgrip: The participant was asked to perform 50% of the maximum voluntary contraction with the right hand for 1 min using a previously calibrated handheld dynamometer (Pertusio and Roatta 2023). To this end, they were provided with visual feedback from the computer monitor, which was continuously displaying the developed force and a horizontal cursor indicating the target force level, set at 50% of the maximum voluntary contraction.
v. Post-handgrip muscle ischemia: Ten seconds before the cessation of the isometric handgrip, a blood pressure cuff (Gima SpA, Milan, Italy), previously wrapped around the right arm, was inflated to 250 mmHg pressure and maintained at this level for 2 min to occlude arterial blood flow. The participant was later asked to rate the peak pain level experienced during the task, using a visual analogue scale.

### Data analysis and statistics

All signals were continuously digitally sampled (CED Micro 1401 acquisition board and Spike2 ver. 9.15, Cambridge Electronic Design, Cambridge, UK) at 100 Hz, and statistical analysis was performed using MATLAB^®^ ver. R2023a (The MathWorks, Natick, MA, USA). The *baseline* values of all variables were obtained as the time average calculated over the 20-s interval preceding the beginning of each task, the task values were calculated over the last 10-s of task execution, and the task effect was calculated as the difference between these two values and indicated with Δ = task–baseline. The Kolmogorov–Smirnov test was used to assess the data’s normal distribution. Student *t* tests were used to assess the statistical significance of stress-induced changes (Δ) in all variables. In addition, the difference between effects on temporal and masseter muscles is also investigated using Hochberg’s test with Dunn/Sidak alpha correction for multiple comparisons. The results are presented as mean ± standard deviation in the text and by box plots in the figures. Finally, the correlation was investigated between individual changes exhibited by TOI (ΔTOI) in the two muscles and between each muscle and the blood pressure change (ΔABP) by the Pearson’s correlation coefficient, for the different tasks. The significance level was set at *p* = 0.05 for all analyses.

Since, for each task the duration was maintained equal in all subjects, average response curves could be obtained by selecting data windows that included the 20-s interval preceding the beginning of the task and a 20-s interval following the end of the task. Tracings from the different subjects were thus aligned with respect to the beginning of the task at *t* = 20 s, *t* = 0 s being the beginning of the time window. Small differences in task duration between subjects might have slightly affected the shape of the curve only after the end of the task.

## Results

**Fig. 1:**
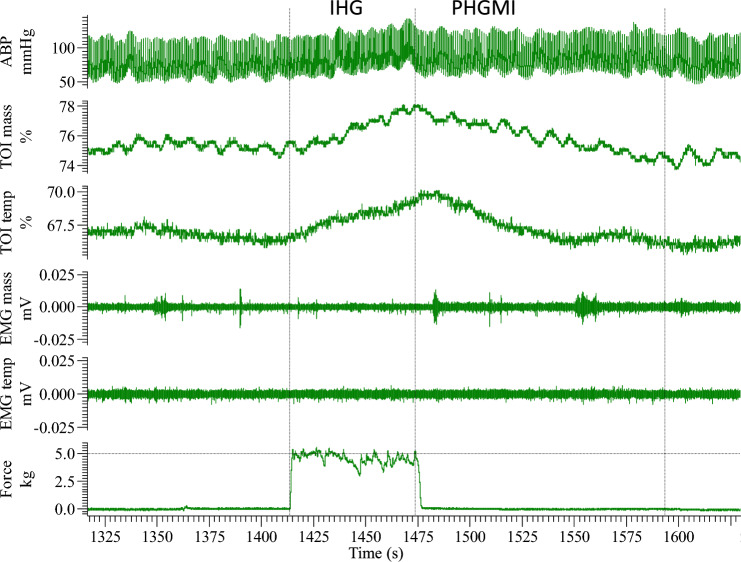
Original recordings from a representative subject showing the response to isometric handgrip (IHG) and post-handgrip muscle ischemia (PHGMI). From top to bottom, arterial blood pressure (ABP), tissue oxygenation index (TOI), electromyography (EMG) from the temporal (temp) and masseter (mass) muscles, and force. Note the minor electromyographic activity occasionally detected in the masseter muscle

**Fig. 2:**
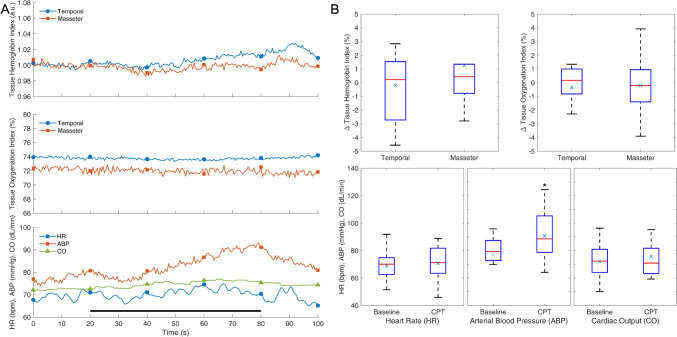
Response to cold pressor test (CPT). Average response curves (**A**) and distribution of effects (**B**) for different variables, as described by mean ( ×), median (red line), interquartile range (blue box), minimum (lower whisker), and maximum (upper whisker) values respectively. The black bar at the bottom indicates the duration of CPT. *p < 0.05; *n* = 14

**Fig. 3:**
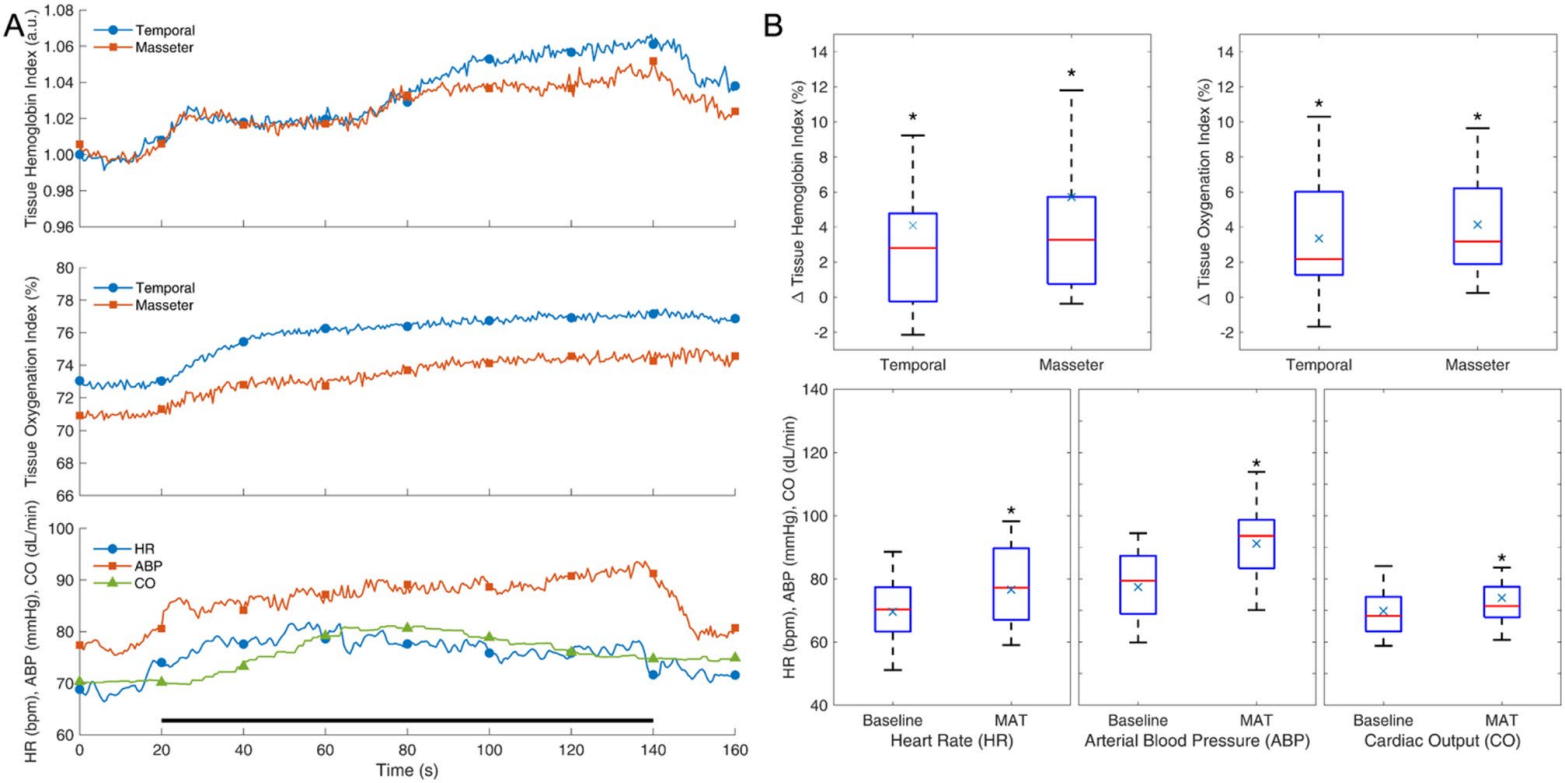
Response to mental arithmetic test (MAT). Average response curves (**A**) and distribution of effects (**B**) for different variables. Notations as in Fig. 2. The black bar at the bottom indicates the duration of MAT. *p < 0.05; *n* = 16

**Fig. 4:**
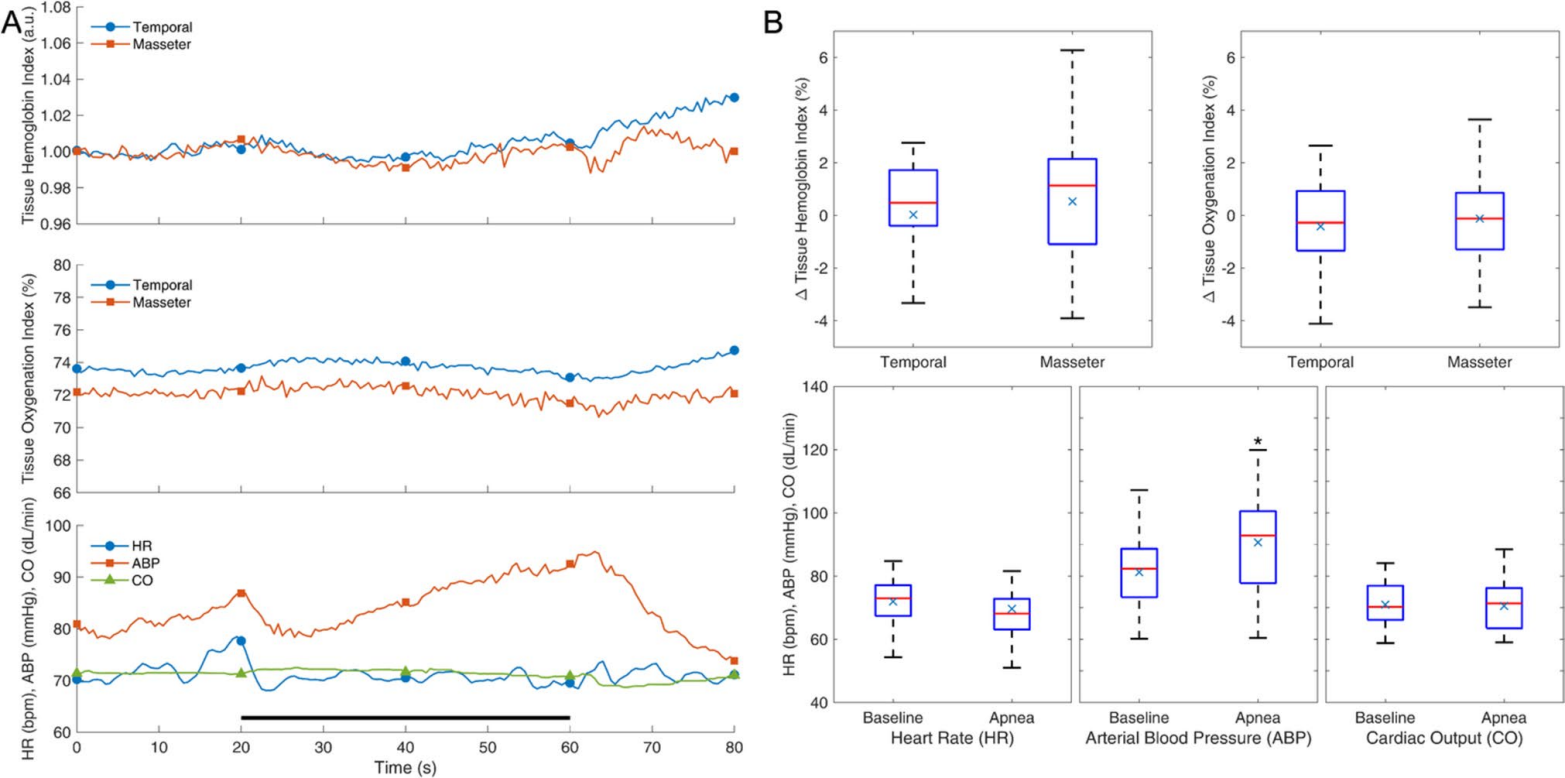
Response to apnea. Average response curves (**A**) and distribution of effects (**B**) for different variables. Notations as in Fig. 2. The black bar at the bottom indicates the duration of apnea. *p < 0.05; *n* = 16

**Fig. 5:**
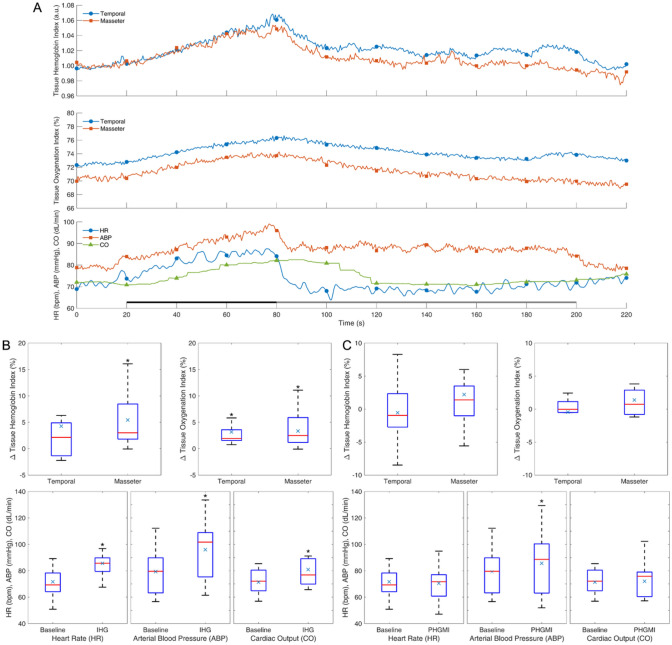
Response to isometric handgrip (IHG) and post-handgrip muscle ischemia (PHGMI). Average response curves (**A**), distribution of effects of IHG (**B**), and PHGMI (**C**) for the different variables. Notations as in Fig. 2. The black and gray bars at the bottom indicate the duration of IHG and PHGMI, respectively. *p < 0.05; *n* = 15

All subjects (excluding two from cold pressor test, one from isometric handgrip, and post-handgrip muscle ischemia) managed to maintain masticatory muscles relaxed both during baseline intervals and stress tests so that no relevant sign of electromyography activation was detected. An example of original recordings is presented in Fig. 1 illustrating the response to isometric handgrip and post-handgrip muscle ischemia in a representative subject. Some spurious and very low (< 15 µV) electromyographic activity is occasionally detected in the masseter muscle with no visible hemodynamic effects. For the different tests, the time course of the response of NIRS and systemic variables is presented, along with the comparison of average effects in temporal and masseter muscles.

i. Cold pressor test: Cold pressor test evoked a self-reported pain score of 7.1 ± 1.8. The hemodynamic response is reported by the average curves of Fig. 2A and the distribution of effects for the different variables is presented in Fig. 2B. The test significantly increased ABP (from 77.5 ± 14.1 to 91.1 ± 18.2 mmHg, *p* < 0.05) while producing a non-significant change in heart rate (from 69.3 ± 10.6 to 71.2 ± 12.0 bpm), cardiac output (from 7.2 ± 1.2 to 7.6 ± 1.8 dL/min) and TOI (temporal muscle: from 72.3 ± 5.0 to 72.0 ± 5.2 %; masseter muscle: from 73.9 ± 6.2 to 73.7 ± 6.8 %). The observed TOI changes in the two muscles were moderately correlated (*r* = 0.42). Finally, we investigated whether the observed vascular reaction (ΔTOI) was correlated to the magnitude of the pressor response (ΔABP): *r* = 0.01 for temporalis and *r* = 0.43 for masseter muscle.
ii. Mental arithmetic test: The average response to the mental arithmetic test (Fig. 3A and B) also exhibited a significant increase in ABP (from 77.9 ± 11.0 to 91.7 ± 11.4 mmHg, *p* < 0.05), with significant changes also in heart rate (from 70.0 ± 10.1 to 77.0 ± 12.2 bpm, *p* < 0.05) and cardiac output (from 7.0 ± 1.1 to 7.4 ± 1.2, *p* < 0.05). Tissue oxygenation and blood volume exhibited a significant increase in both temporal (TOI: from 71.3 ± 6.9 to 74.8 ± 7.0 %; ΔTHI: 0.04 ± 0.06 %, *p* < 0.05) and masseter (TOI: from 72.8 ± 6.1 to 77.0 ± 4.9 %; ΔTHI: 0.06 ± 0.08, *p* < 0.05) muscles. The observed TOI changes in the two muscles exhibited strong correlation (*r* = 0.70) but poor correlation with ABP changes (r < 0.23).
iii. Apnea: The response to apnea is reported by the average curves of Fig. 4A and the distribution of effects for the different variables is presented in Fig. 4B. The test significantly increased ABP (from 81.7 ± 13.2 to 91.1 ± 14.7 mmHg, *p* < 0.05) while producing a non-significant effect in heart rate (from 72.4 ± 9.7 to 70.1 ± 12.3 bpm), cardiac output (from 7.14 ± 0.7 to 7.10 ± 0.8 dL/min) and NIRS variables (temporal muscle TOI: from 72.1 ± 7.0 to 71.7 ± 6.4 %; masseter muscle TOI: from 73.5 ± 5.8 to 73.4 ± 5.6 %). The observed TOI changes in the two muscles exhibited strong correlation (*r* = 0.70) and a moderate negative correlation with ΔABP: r = − 0.48 for temporalis and *r* = − 0.34 for masseter muscle.
iv. Isometric handgrip and post-handgrip muscle ischemia: The response to isometric handgrip and post-handgrip muscle ischemia is presented by the average curves in Fig. 5A and the distribution of effects in Fig. 5B and C for the different variables. Isometric handgrip provoked a significant increase in ABP (from 79.8 ± 18.0 to 96.4 ± 21.8 mmHg, *p* < 0.05), heart rate (from 72.1 ± 11.1 to 86.1 ± 11.5 bpm, *p* < 0.05) and cardiac output (from 7.2 ± 0.9 to 8.1 ± 1.6, *p* < 0.05).
  Tissue oxygenation increased in both temporal (from 70.5 ± 6.2 to 73.7 ± 6.9 %, *p* < 0.05) and masseter (from 72.5 ± 6.1 to 76.0 ± 5.6 %, *p* < 0.05) muscles, while THI increased only in masseter muscle (ΔTHI: 0.06 ± 0.05 %, *p* < 0.05). The observed TOI changes in the two muscles exhibited strong correlation (*r* = 0.78), and poor correlation with ΔABP (*r* < 0.25).
  Compared to resting levels (before isometric handgrip), post-handgrip muscle ischemia exhibited a significant increase in ABP (from 79.8 ± 18.0 to 86.1 ± 22.4 mmHg, *p* < 0.05) while producing non-significant effects in heart rate (from 72.1 ± 11.1 to 71.0 ± 13.0 bpm) and cardiac output (from 7.2 ± 0.9 to 7.3 ± 1.3). Also, NIRS variables did not exhibit significant changes in temporal (TOI: from 70.5 ± 6.2 to 70.1 ± 8.0 %) and masseter (TOI: from 72.5 ± 6.1 to 74.0 ± 6.8 %) muscles. The observed TOI changes in the two muscles exhibited moderate correlation (*r* = 0.60), and strong negative correlation with ΔABP (*r* < − 0.7). Maximum pain score during post-handgrip muscle ischemia was: 6.6 ± 1.8.

## Discussion

The results described hemodynamic responses of temporalis and masseter muscles to different stressors, which effectively increased sympathetic activity, as indicated by the significant increase in ABP in some cases accompanied by an increase in heart rate and cardiac output. In particular, tissue oxygenation significantly increased in both muscles during mental arithmetic test and isometric handgrip, accompanied by increased or unchanged blood volume which indicates increased muscle perfusion during these tests (Boushel and Piantadosi 2000; Fadel et al. 2004; Rashid and Roatta 2022). No significant changes were instead observed during cold pressor test, apnea, and post-handgrip muscle ischemia. However, none of the tested conditions provided evidence of sympathetic-induced vasoconstriction. Both temporalis and masseter muscles consistently exhibited similar response patterns in all tested conditions.

It is well known that sympathetic activation may result in skin vasodilation in the facial area (Vassend and Knardahl 2005). A number of NIRS studies also reported dilatory responses (increased oxygenation) in the masseter muscle in response to certain stress stimuli, e.g., cold pressor test (Maekawa et al. 1998), and mental calculation (Hidaka et al. 2004), although classical methodologies did not discriminate skin from muscle contribution (Grassi and Quaresima 2016). As discussed in more detail below, this can be achieved by techniques such as spatially-resolved spectroscopy which effectively excludes contribution from the skin (Messere and Roatta 2013) and recently allowed us to prove that several types of stress result in specific dilatation of the masseter muscle, in contrast to limb muscles where marked vasoconstriction may take place (Rashid and Roatta 2022). This latter study was the first to directly compare hemodynamic stress responses in head muscles and included an extensive review of the recent literature, although head muscles were limited to the masseter muscle (Rashid and Roatta 2022). Limb muscles were no longer examined in the present study but sympathetic constrictory responses or increases in muscle sympathetic nerve activity in these muscles have consistently been reported in response to several stressors, including cold pressor test (Wray et al. 2007; Jacob et al. 2021; Coovadia et al. 2022), post-exercise muscle ischemia (Saito et al. 2000; Tokizawa et al. 2006), and acute pain stimuli (Burton et al. 2016).

The results of the present study confirm the different pattern of head muscles, now including the temporalis muscle. We are not emphasizing the dilatory responses observed during mental arithmetic test and isometric handgrip, as these are known to take place in limb muscles as well (Rusch et al. 1981; Carter et al. 2005; Rashid and Roatta 2022). Unexpectedly, the significant TOI increase in response to cold pressor test and post-handgrip muscle ischemia previously observed for the masseter muscle (Rashid and Roatta 2022) was no longer observed in the present study. While the absence of response to cold pressor test could be partly attributed to the milder stimulus intensity, in terms of water temperature (10 instead of 8 °C) and duration (1 instead of 2 min), the response to post-handgrip muscle ischemia has no apparent explanation except that the subject position was sitting instead of supine. Incidentally, both active and passive dilatory responses have found to be attenuated above as compared to below heart level (Trinity et al. 2011; Jasperse et al. 2015; Seddone et al. 2020). It is possible that the unfavorable hydrostatic gradient affecting the head region or the increased sympathetic tone have attenuated the hyperemic response in the sitting position, compared to the supine. In fact, in human skeletal muscles, competing vascular actions are exerted by the dilatory action of circulating adrenaline and the constrictory action of the neurally released noradrenaline (Montoya et al. 1997; Terakawa and Ichinohe 2012). In this respect, it is interesting to observe that a poor positive correlation was observed between ΔTOI and ΔABP in mental arithmetic test and isometric handgrip; while a strong negative correlation resulted in post-handgrip muscle ischemia. This observation fits with the concept of prevailing adrenaline secretion during mental arithmetic test and isometric handgrip (Goldberg et al. 1996; Joyner and Dietz 2003; Rashid and Roatta 2022) and prevailing noradrenaline constriction in post-handgrip muscle ischemia: the correlation is poor in the first case; whereby ABP is largely mediated by the increase in cardiac output (significantly increased in these tests). On the contrary during post-handgrip muscle ischemia, ΔABP is mainly mediated by the increased vasoconstriction, being cardiac output unchanged or even decreased (Rashid and Roatta 2022). On this basis, it is plausible to speculate that the baroreflex-mediated increase in sympathetic vasconstrictory tone, associated with the sitting position, has shifted the balance and attenuated the stress-related dilatory responses, compared to the supine position. Dedicated studies re-testing the responses to the same stressor in the same subjects in the two body postures may be designed to test this hypothesis.

Although simultaneous monitoring of a limb muscle was not performed in this study, a relevant outcome is that temporalis and masseter muscle exhibit consistently similar responses to the variety of employed stressors. In fact, this observation supports the hypothesis put forward in our previous study that muscle blood flow is differentially controlled in limb and head muscles. Very few studies investigating differences in blood flow control of lower and upper limb muscles evidenced stronger sympathetic constrictor effects in lower limbs (Eklund and Kaijser 1976; Rusch et al. 1981), we speculate that the constrictory sympathetic action is modulated according to the hydrostatic gradients of the body with erect posture, i.e., the constrictory action is stronger where hydrostatic load and blood pressure are higher. Whether this effect is mediated by differences in sympathetic neural drive and/or differences in the density and distribution of adrenergic receptors subtypes remains to be investigated.

A major limitation of classical NIRS measurement, based on the modified Beer–Lambert methodology is that the contribution from superficial cutaneous layers cannot be discriminated the contribution from deeper layers, e.g., brain or muscle. Spatially-resolved spectroscopy was proven to be particularly effective in focusing the measurement in deep layers as documented in several studies for both cerebral (Canova et al. 2011; Moerman et al. 2022) and muscle monitoring (Messere and Roatta 2013; Grassi and Quaresima 2016). In particular, we showed that contrary to standard NIRS, spatially-resolved spectroscopy was neither affected by local increases in cutaneous microcirculation (Messere and Roatta 2013) nor by increased blood flow in superficial veins (Messere and Roatta 2015), provoked by warming of local or remote distal areas, respectively. For this reason, spatially-resolved spectroscopy was adopted in this study. Besides excluding interference from the skin, NIRS monitoring of the temporal muscle also requires to exclude possible contributions from the deeper cerebral tissue. However, the depth of the sample volume is proportional to the inter-optode distance (about 50% of it). In a previous methodological study, we showed that reducing the inter-optode distance from 4 to 3 cm effectively prevented the influence of cerebral hemodynamic changes, as produced by hyperventilation, on NIRS monitoring of the temporal muscle (Rashid and Roatta 2023). Though, that study also evidenced the placement of the NIRS probe could be critical in some subjects, probably due to the fact that the temporalis muscle becomes too thin and superficial under the hair-free area of the temple, in which case spatially-resolved spectroscopy measurements would not adequately sample the muscle tissue, thus resulting in possible underestimation of the hemodynamic response. We cannot exclude that this could have happened also in the present study. In fact, we observed in a few subjects that changes in tissue oxygenation were considerably weaker than changes in oxygenated and deoxygenated hemoglobin.

An additional limitation of this study is the low sample size, which prevented analysis of sex-related differences. Considered the sex-related prevalence of clinical manifestations such as headache and temporomandibular disorders, the issue is worth to be addressed in future investigations. Finally, we recruited the subjects from a young and healthy student population, we did not assess psychological traits that could affect the reactivity to the different stressor, although this relation this not trivial; e.g., with regard to the pain stress, it has been shown that the autonomic activation is psychogenic in nature, but its variability among individual appeared to be uncorrelated with the anxiety score (Burton et al. 2016).

To conclude, head muscles appear to be similarly controlled by the autonomic nervous system under exposure to different types of stress and generally protected from stress-induced hypoperfusion. From a finalistic point of view, this could be meant to favor activity of jaw muscles, being frequently involved in aggressive/defensive behavior in different animal species. Protection from hypoperfusion may also be beneficial against the development of temporomandibular joint dysfunction (Delcanho 1995; Maekawa et al. 2002; Exposto et al. 2021). It should however be noted that these considerations are based on average responses and that clear vasoconstrictive responses were occasionally observed in some subject, possibly depending on a different balance between beta- and alpha-adrenergic vascular control. In addition, sympathetic reactivity to stress is known to present individual variability related to different factors including, sex, age, the nature and duration of the stressful stimulus (Greaney et al. 2015; Burton et al. 2016; Coovadia et al. 2022). Whether the susceptibility to temporomandibular joint disorders (such as myofascial orofacial pain and temporal headache) development depends on the individual hemodynamic responsiveness to stress needs to be ascertained.

## Data Availability

The data that support the findings are available from the corresponding author upon reasonable request.

## Abbreviations

NIRS: Near-infrared spectroscopy
TOI: Tissue oxygenation index
THI: Tissue hemoglobin index
ABP: Arterial blood pressure
